# Non-Linear Conductivity Epoxy/SiC Composites for Emerging Power Module Packaging: Fabrication, Characterization and Application

**DOI:** 10.3390/ma13153278

**Published:** 2020-07-23

**Authors:** Rui Li, Yufan Wang, Cheng Zhang, Hucheng Liang, Jin Li, Boxue Du

**Affiliations:** 1 National Quality Supervision & Inspection Center of Electrical Equipment Safety Performance, Zhejiang Huadian Equipment Testing Institute, Hangzhou 310015, China; 13958020182@163.com; 2 School of Electrical and Information Engineering, Tianjin University, Tianjin 300072, China; 15122557071@163.com; 3 State Grid Jiangsu Electric Power Co., Ltd., Maintenance Branch Company, Nanjing 211102, China; peter.c.zhang@aliyun.com

**Keywords:** non-linear conductivity, epoxy/SiC composites, crystal morphology, temperature, power module packaging

## Abstract

In this paper, SiC/epoxy resin composites containing different amounts of micro-sized SiC with different crystal morphologies were fabricated to study the effects of crystal morphology and temperature on non-linear conductivity characteristics. The research results illustrate that the *β*-SiC particles can provide a higher non-linear conductivity, compared with the *α*-SiC particles. The presence of temperature also affected the non-linear conductivity behaviors of the epoxy/SiC composites. When the *α*-SiC content was low, the non-linear conductivity coefficient of the composites increased rapidly as the temperature increased, but the non-linear conductivity decreased slightly as the temperature increased when the filler concentration was large enough. To reduce the influence of the electric field concentration effect by the increase in power density on the power module packaging, the voltage sharing application of the SiC/epoxy composites was simulated by COMSOL Multiphysics (v5.2a, COMSOL Inc., Stockholm, Sweden). The results show that the composites with non-linear conductivity can reduce the electric field stress. The emerging insulation material obtained by the SiC-modified epoxy resin can effectively promote electric field distribution uniformity, and ensure the safe operation of the power module.

## 1. Introduction

In recent years, epoxy resin composites have been widely applied in insulation equipment and module packaging, due to their exceptional performance including good heat resistance properties, excellent mechanical properties, and great insulating properties [1,2,3]. As the basic material of electronic packaging, epoxy resin composites affect the performance of the packaging. The development of integrated circuits has promoted the development of module packaging in the direction of miniaturization, lightness and thinness, and an increase in power density with the volume reduction bringing great challenges to epoxy resin material for power module packaging [4]. The current power module packaging requires good insulating properties of epoxy resin composites under a high electric field and at a high temperature.

Inorganic fillers with non-linear conductivity characteristics have been introduced into the polymer matrix to obtain non-linear conductivity composites. Many studies have shown that the above-mentioned composites can intelligently regulate conductivity characteristics to make the electric field uniformly distributed, because the conductivity of materials is highly dependent on the electric field strength as the electric field exceeds a certain value [5,6,7,8]. Moreover, the non-linear conductivity enables composites to suppress space charge accumulation and electric branch growth, thereby effectively improving the insulation performance of the equipment [9,10].

Many researchers focused on non-linear conductivity composites. Varlow et al. [11] studied the epoxy composites with ZnO particles. It was found that the composites showed obvious non-linear conductivity behaviors when the volume fraction of ZnO fillers reached 14%. He et al. [12] found that the SiR/ZnO composites can make the electric field uniformly distributed at the tip and suppress corona discharge. Nelson et al. [13] introduced the SiC particles into silicone rubber polymer to improve the electric field distribution. Wang et al. [14] introduced graphene oxide into polymer and provided great non-linear conductivity at a filler loading of 3%. The presence of temperature gradients in the dc insulation system also substantially affected the conductivity distribution of the polymer, and the electric field distribution was distorted. Imai et al. [15] studied the electrical performance of non-layered silicate-containing epoxy composites, and the changing trend of volume resistivity with temperature. The results conclude that the resistivity of the composites decreases linearly as the temperature increases, and at the same temperature, the resistivity of the non-layered silicate/epoxy composites is lower than that of the neat epoxy resin. Maryam et al. [16] applied a non-linear field-dependent conductivity (FDC) layer to wide bandgap power modules, in order to study the effects of temperature on the electric field distribution optimization method. The results indicate that the electric field reduction method employing the non-linear FDC layer is almost temperature-independent. The non-linear conductivity composites have been widely used in cable accessories and wall bushing flanges, effectively improving the insulation performance of the materials. The ethylene propylene diene monomer rubber (EPDM) with non-linear conductivity properties has been applied in dc cable accessories to suppress the electric field distortion [17]. However, the effect of SiC on the insulating performance of epoxy resin composites with various crystal morphologies at different temperatures is not clear, and there are few studies about the effect of emerging epoxy/SiC composites on power module packaging.

In this paper, epoxy resin composites containing different amounts of SiC with various crystal morphologies were prepared. Then, the effects of crystal morphology and temperature on non-linear conductivity characteristics were studied. Furthermore, the application of voltage sharing in the typical structure of power module packaging was simulated by COMSOL Multiphysics.

## 2. Materials and Methods

### 2.1. Sample Preparation

To obtain the epoxy/SiC composite samples, the bisphenol A epoxy resin was selected as the material matrix, the low molecular weight polyamide resin hardener was selected as the curing agent, and two types of micro-sized SiC (*α*-crystal and *β*-crystal, ~12 μm diameter, density of 3.2 g/cm^3^) were selected as fillers. The epoxy resin matrix used in this experiment is a high-activity, low-viscosity liquid epoxy resin, which has good electrical insulation and wettability. The curing agent polyamide resin HY-165 can be mixed with epoxy resin and various additives to obtain products for various applied conditions; in particular, it can cure the epoxy resin at room temperature or heating conditions without by-products, and the cured product performance is better than that of general amine or anhydride curing agent. Table 1 lists the epoxy resin technical index, and Table 2 lists the curing agent technical index.

**Figure 1:**
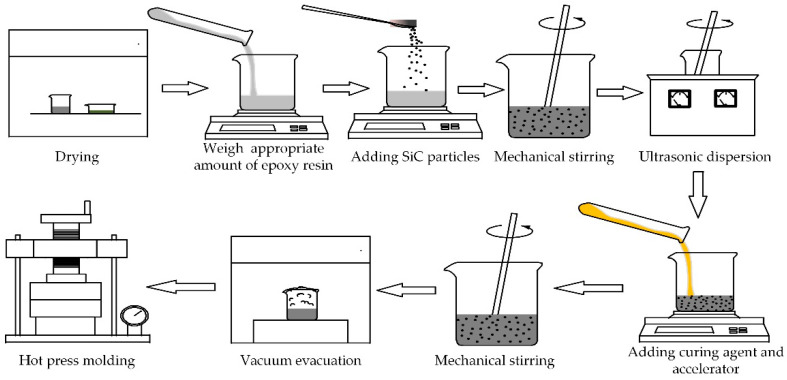
Flow chart of sample preparation of the epoxy resin/SiC composites.

Figure 1 shows the flow chart of the sample preparation. The pre-dried micro-SiC was dispersed into the epoxy matrix, and the mixture was stirred for 40 min, and then ultrasonicated for 40 min to make the SiC particles disperse uniformly in the epoxy matrix. Next, the hardener was doped into the mixture and churned for 20 min. Then, vacuum evacuation was performed to avoid bubbles in the mixture. Finally, the mixture was poured into a pre-heated tablet press mold and cured for 15 min. The pressure and temperature of the tablet press were set to 10 MPa and 120 °C, respectively. After the mold was naturally cooled to room temperature, the epoxy/SiC composite samples were obtained. The samples prepared in this experiment were square samples of 9 × 9 cm^2^, with a thickness of 0.5 mm.

In this experiment, epoxy/*α*-SiC composite samples with *α*-SiC content of 60, 90, 120, and 150 phr were prepared; the epoxy/*β*-SiC composite samples were prepared with *β*-SiC content of 60, 90, and 120 phr. The contents were expressed as a percentage by weight, that is, 60 phr means that the epoxy resin matrix was 100 g, and the SiC fillers were 60 g in weight. The conversion of the corresponding mass fraction and volume fraction is shown in Table 3.

### 2.2. Characterization Measurement

X-ray diffraction (XRD) is a traditional analytical method used to study the difference in the crystal morphology structure of the SiC fillers. The microstructural morphology characteristics of the composite samples were tested using a scanning electron microscope (SEM, FEI, Hillsboro, OR, USA) to study the dispersion situation of particles in the composites.

### 2.3. Conductivity Measurement

The three-electrode experiment system was used to measure the volume conductivities of the samples [18]. The measurement system mainly included a three-electrode unit, a protection resistance, a dc source, and an electrometer Keithley 6517B (Tianjin, China). It is noted that the whole system needed to be placed in a constant temperature and humidity chamber to improve the measurement accuracy and prevent the influence of temperature fluctuation on the measurement data. The tested sample should be wiped with alcohol to remove the impurities remaining on the sample surface before the measurement. The current *I* was continuously detected for 35 min, and the average value of the measured current in the last two minutes was taken as the conductivity current of the tested sample.

The conductivity *σ* of the sample can be calculated by the formula:

(1) $$\sigma =\frac{I}{U}\cdot \frac{4L}{\pi {\left(d+g\right)}^{2}}$$

where *U* is the experimental voltage applied to the samples, *L* is the thickness of the samples, *d* is the diameter of the measurement electrode, 50 mm, and *g* is the distance between the measurement electrode and the ring-shaped electrode, 2 mm.

The non-linear characteristic between *I* and *U* can be expressed as:

(2) $$I=KU^{\alpha }$$

where *K* is the coefficient related to polymer and structural parameters, and *α* is the non-linear coefficient of the polymer. The *J–E* equation can be obtained by converting the above Equation (2):

(3) $$J=K_{1}E^{\alpha -1}$$

where *K*_1_ is the constant related to material properties. The relationship between the conductivity *γ* and the electric field strength *E* is:

(4) $$\gamma =AE^{k}$$

where *k = α − 1* is the non-linear coefficient.

By taking the logarithm of Equation (4), the *γ–E* relationship can be obtained:

(5) lgγ=lgA+klgE

The non-linear conductivity of the composites can be described by the *J–E* relationship between the current density *J* and the electric field *E*, and the non-linear coefficient *k* can be calculated by the next equation:

(6) $$k=\frac{\mathrm{lg}(J_{1}/J_{2})}{\mathrm{lg}(E_{1}/E_{2})}$$

where the current density *J*_1_, *J*_2_ is corresponding to the electric field *E*_1_, *E*_2_, respectively, and usually the value of *J*_1_/*J*_2_ is greater than 10.

## 3. Results and Discussion

### 3.1. Characterization Analysis

**Figure 2:**
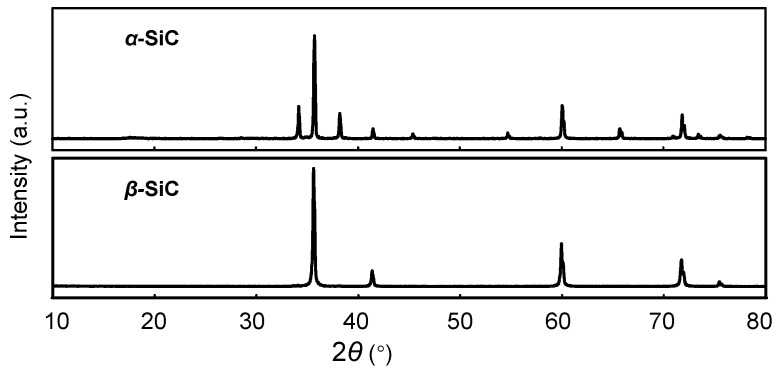
X-ray diffraction results of the SiC particles.

**Figure 3:**
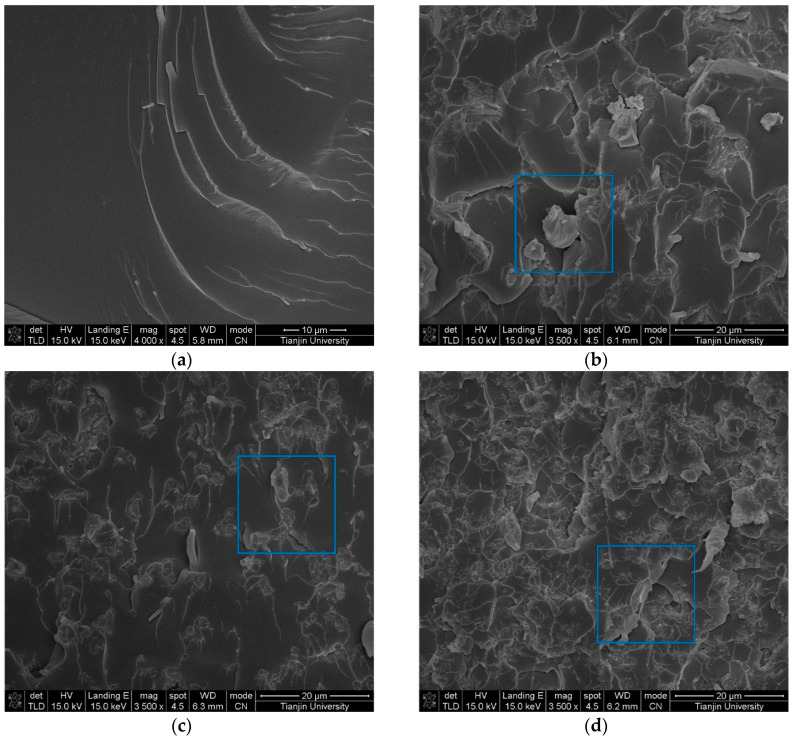
Scanning electron microscope (SEM) images of the epoxy composites filled with SiC particles of different filler content. (**a**) SiC of 0 phr, (**b**) *α*-SiC of 90 phr, (**c**) *β*-SiC of 60 phr, (**d**) *β*-SiC of 120 phr.

As shown in Figure 2, the XRD results indicate that the structure of *α*-SiC is a hexagonal crystal, and the structure of *β*-SiC is a cubic crystal, according to the standards document. Figure 3 shows SEM images of the epoxy composites filled with SiC particles of different filler content. It can be observed that the SiC particles are evenly dispersed in the epoxy matrix. When the concentration of SiC is low, the SiC particles disperse in the matrix, and there is no contact between the particles. When increasing the SiC content, contact between the particles occurs, and the percolation path begins to form. A perfect permeation path was formed in composite with a high SiC filler content.

### 3.2. Effects of Crystal Morphology

The dc volt-ampere characteristics at room temperature (20 °C) were measured, and the non-linear coefficient *k* and the threshold field *E*_b_ were calculated, according to the measurement results.

**Figure 4:**
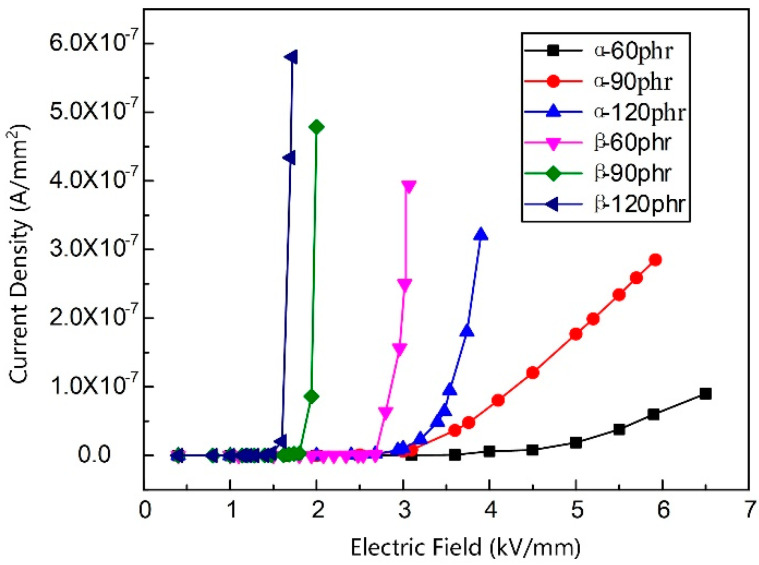
*J–E* curves of the epoxy/SiC composite samples with different SiC crystal morphologies.

Figure 4 shows the dc conductivity volt-ampere characteristic curves of the epoxy/SiC composite samples with different crystal morphologies. As shown in Figure 3, the samples with different crystal morphologies have distinct non-linear conductivity characteristics. The current density of the samples increases by an order of magnitude when the electric field strength exceeds the threshold field. The current density of the epoxy/*β*-SiC composite increases more rapidly than that of the epoxy/*α*-SiC composite sample, and accordingly, the electric field strength of the epoxy/*α*-SiC composite sample is smaller than that of the epoxy/*β*-SiC composite sample, after the non-linear characteristics appear.

In Table 4 and Table 5, the non-linear coefficient *k* and the threshold field *E*_b_ of samples with different *α*-SiC and *β*-SiC contents are acquired, respectively. The non-linear coefficient of the epoxy/SiC composite samples shows an upward trend, and the threshold field shows a downward trend with the increase in filler content. The non-linear coefficient of the epoxy/*β*-SiC composite is significantly greater than that of the epoxy/*α*-SiC composite with the same filler content. In particular, when the SiC content is 60 phr, the non-linear coefficient of the epoxy/*β*-SiC composite reaches 30.22, which is nine times more than that of the *α*-SiC composite with the same filler content.

### 3.3. Effects of Temperature

To study the impact of temperature on the conductivity of the epoxy/SiC composites, the dc volt–ampere characteristics of the composite samples with *α*-SiC content of 60 and 150 phr were measured at 20, 40, 60 and 80 °C, respectively, and the non-linear coefficient *k* and the threshold field *E*_b_ were calculated.

**Figure 5:**
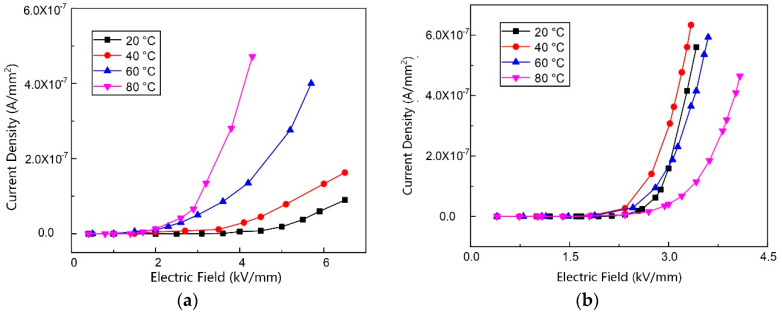
*J–E* curves of the epoxy/SiC composite samples at different temperatures with a filler content of (**a**) 60 and (**b**) 150 phr.

Figure 5 presents the dc conductivity volt–ampere characteristic curves of the composite samples at different temperatures. Figure 5a,b present the experimental results of samples containing 60 and 150 phr of *α*-SiC, respectively. Table 6 and Table 7 are the non-linear coefficient *k* and the threshold field *E*_b_ of two samples at different temperatures. When the SiC content is low (60 phr), the current density of the composite sample increases with the rising temperature under the same electric field, while the non-linear coefficient increases and the non-linear threshold field drops. When the SiC content reaches 150 phr, the current density at 20, 40, and 60 °C is relatively close under the same electric field stress. Generally, different from the former composites with a low filler content, the non-linear conductivity of composites with a high filler content decreases slightly with the increasing temperature.

### 3.4. Discussion

The crystal morphology can affect the non-linear conductivity behaviors of epoxy/SiC composites. The *β*-SiC (rod-shaped) is easier to form a percolation path than that of *α*-SiC (granular-shaped), as the electric field strength reaches the threshold field, which increases the non-linear conductivity of the composites [19,20]. In addition, the crystal structure causes differences in conductivity between different crystal morphologies. The array of tetrahedra parallel bonded layers in the *β*-SiC structure results in a regular arrangement sequence and gaps between internal vents, meaning that more voids go into action as the voltage increases, and the non-linear conductivity is greater than that of the *α*-SiC [21].

The temperature can also affect the non-linear conductivity behaviors of epoxy/SiC composites. When the filler content is low, the SiC particles in the epoxy/SiC composite do not contact with each other to form a percolation path, and only a small part of the carriers participate in the hopping conduction process, making the conductivity increase slowly with the enhancement of the electric field. When the temperature rises, the carrier concentration, as well as the thermal excitation process of the carriers, is increased obviously, leading to the rapid increase in non-linear conductivity [13,22]. On the other hand, as a result of a relatively narrow forbidden bandgap of SiC between the valance band and the conduction band, leading the carriers easily excited to the conduction gap by heating, and increasing the conductivity [23]. When the filler content reaches a certain high level, a perfect percolation path is formed in the composite bulk, and the transportation of charges through the percolation path of the SiC sharply increases the conductivity. When the temperature increases, the conductivity of epoxy resin shows a “positive temperature coefficient effect”; that is, the conductivity of the epoxy resin matrix obviously increases as the operating temperature rises [24], while the conductivity of the SiC decreases with the temperature. The competition between the two results in a slight decrease in non-linear conductivity with the increase in temperature.

## 4. Application

The typical module packaging structure was given in [25,26,27]. The Insulated Gate Bipolar Transistor (IGBT) and diodes were welded on a packaging substrate composed of two metallization layers and a ceramic layer, and the other side of the metalized substrate was brazed on the base plate. The entire module was encapsulated with an insulating gel that can protect semiconductors, substrates, and connections from moisture, vibration, and discharge.

**Figure 6:**
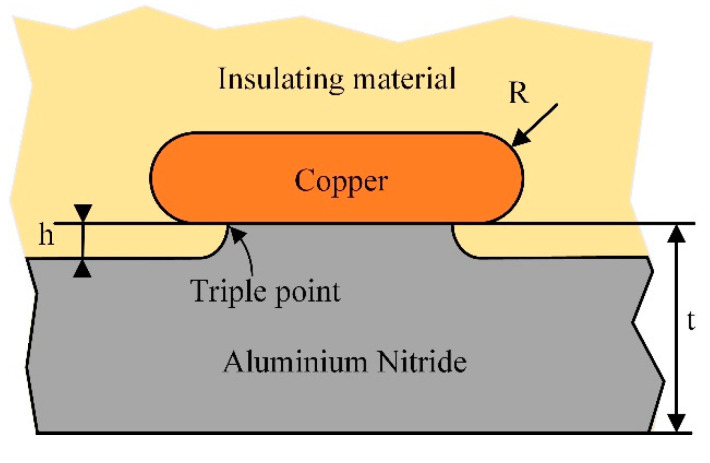
Protruding structure.

**Figure 7:**
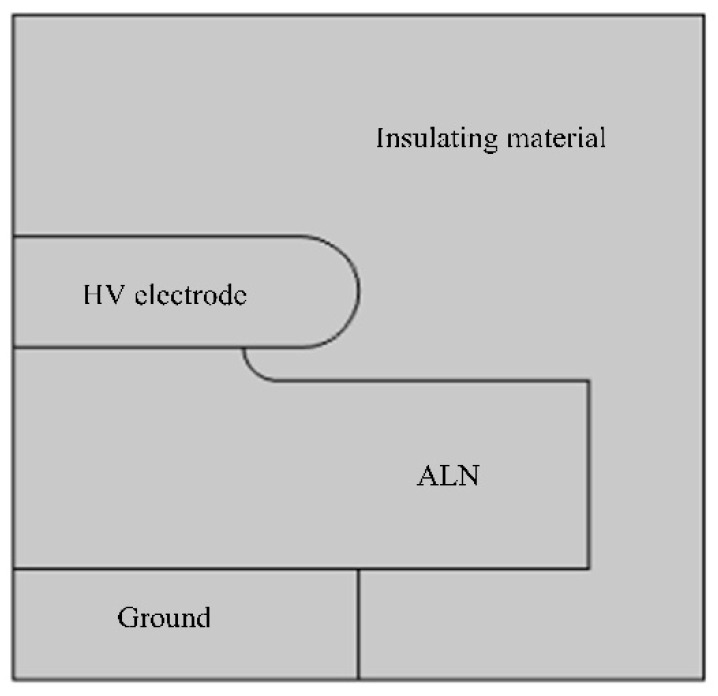
Simulation model.

The protruding substrate of the module is shown in Figure 6 [28]. A 500 μm thick copper electrode, whose radius of curvature is R = 250 μm, was soldered on the top of a 1 mm thick AlN protruding substrate [25]. The welding point had a 150 μm tall protrusion. To study the application of the epoxy/SiC composites with non-linear conductivity in power module packaging, a simulation model by COMSOL Multiphysics is shown in Figure 7. The above-obtained conductivity parameters *σ*(*E*) were used to equalize the electric field distribution, and the neat epoxy material without the SiC fillers was used as a comparison to investigate the optimization effect. Table 8 lists the partial electrical parameters of the materials used in the simulation. A 4.5 kV dc voltage was applied to the high voltage (HV) electrode, and the other electrode was grounded.

**Figure 8:**
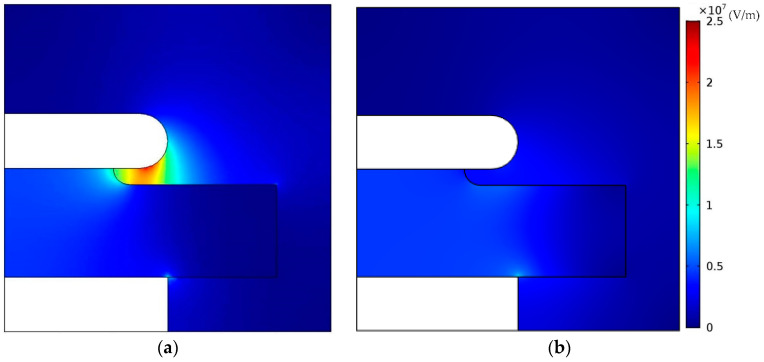
The electric field distribution of power modules for applying (**a**) neat epoxy material; and (**b**) non-linear conductivity epoxy/SiC composite.

The electric field strength distribution of power modules with the neat epoxy material and the epoxy/SiC composite as the insulating materials is presented in Figure 8. The composites with non-linear conductivity can observably weaken the intensified electric field stress at the triple point, compared with that of neat material. The maximum electric field of module packaging reduces from 24.4 to 10.2 kV/mm, which means that the electrical field of module packaging has a uniform trend, hence the epoxy/SiC composites can lead to a uniform electric field distribution.

## 5. Conclusions

The epoxy resin composites containing different amounts of SiC with different crystal morphologies were prepared, and crystal morphology and temperature were found to affect the non-linear conductivity characteristics. The electric field strength distribution of the module packaging is optimized by the epoxy/SiC composites. The epoxy/SiC composites can regulate the electric field distribution and relax the electric field distortion. The main conclusions are as follows:

- The non-linear conductivity of the epoxy/*β*-SiC composite is significantly greater than that of the epoxy/*α*-SiC composite as the electric field reaches the threshold field.
- The non-linear conductivity of the composites rapidly rises with the rising temperature under the condition of a low *α*-SiC content. However, when the filler content reaches a certain high level, the non-linear conductivity decreases slightly with the enhancement of temperature.
- The epoxy/SiC composites with non-linear conductivity were applied for module packaging using COMSOL Multiphysics. A uniform electric field distribution was obtained, especially at the triple point, and the maximum electric field reduced from 24.4 to 10.2 kV/mm.

## Figures and Tables

**Table 1:** Epoxy resin technical index.

Item	Index
Appearance	Colorless clear liquid
Color (number)	≤ 2
Density (g/mL, 25 °C)	1.02–1.11
Viscosity (MPa, 25 °C)	100–500
Epoxide equivalent (g/eq)	178–195
Shear strength	≥10

**Table 2:** Curing agent technical index.

Item	Index
Appearance Density (kg/m^3^)	Light yellow viscous liquid 0.99
Viscosity (mPa·s, 25 °C) Amine value (mg KOH/g)	200–1000 400

**Table 3:** The unit conversion of the SiC content in the epoxy resin/SiC composites.

SiC Content (phr)	60	90	120	150
**wt%**	37.50	47.37	54.55	60.00
**vol%**	18.22	25.05	30.82	35.78

**Table 4:** The non-linear coefficient *k* and the threshold field *E*_b_ of the samples with different *α*-SiC contents.

*α*-SiC	60	90	120
*k*	3.12	5.56	11.55
*E*_b_ (kV/mm)	4.32	3.36	3.15

**Table 5:** The non-linear coefficient *k* and the threshold field *E*_b_ of the samples with different *β*-SiC contents.

*β*-SiC	60	90	120
*k*	30.22	44.76	45.28
*E*_b_ (kV/mm)	2.83	1.96	1.64

**Table 6:** The non-linear coefficient *k* and the threshold field *E*_b_ of the sample at different temperatures with *α*-SiC content of 60 phr.

Temperature (°C)	20	40	60	80
*k*	3.12	3.66	3.56	4.67
*E*_b_ (kV/mm)	4.32	3.50	3.28	2.66

**Table 7:** The non-linear coefficient *k* and the threshold field *E*_b_ of the sample at different temperatures with *α*-SiC content of 150 phr.

Temperature (°C)	20	40	60	80
*k*	11.73	8.84	7.91	7.95
*E*_b_ (kV/mm)	2.67	2.55	2.83	3.02

**Table 8:** Electrical parameters used in the simulation.

Material	Relative Permittivity	Conductivity *σ* (S/m)
Ceramic substrate	9	10^−11^
Neat epoxy material Epoxy/SiC Composite	3.6 10	10^−15^ *σ*(E)
